# COVID-19 Pandemic Spurs Medical Telerobotic Systems: A Survey of Applications Requiring Physiological Organ Motion Compensation

**DOI:** 10.3389/frobt.2020.594673

**Published:** 2020-11-09

**Authors:** Lingbo Cheng, Mahdi Tavakoli

**Affiliations:** ^1^College of Control Science and Engineering, Zhejiang University, Hangzhou, China; ^2^Department of Electrical and Computer Engineering, University of Alberta, Edmonton, AB, Canada

**Keywords:** COVID-19, healthcare, physical distancing, teleoperation, telerobotics, telemedicine, motion compensation, robot control

## Abstract

The coronavirus disease 2019 (COVID-19) pandemic has resulted in public health interventions such as physical distancing restrictions to limit the spread and transmission of the novel coronavirus, causing significant effects on the delivery of physical healthcare procedures worldwide. The unprecedented pandemic spurs strong demand for intelligent robotic systems in healthcare. In particular, medical telerobotic systems can play a positive role in the provision of telemedicine to both COVID-19 and non-COVID-19 patients. Different from typical studies on medical teleoperation that consider problems such as time delay and information loss in long-distance communication, this survey addresses the consequences of physiological organ motion when using teleoperation systems to create physical distancing between clinicians and patients in the COVID-19 era. We focus on the control-theoretic approaches that have been developed to address inherent robot control issues associated with organ motion. The state-of-the-art telerobotic systems and their applications in COVID-19 healthcare delivery are reviewed, and possible future directions are outlined.

## Introduction

On January 30, 2020, the World Health Organization (WHO) officially declared the coronavirus disease 2019 (COVID-19) outbreak as a public health emergency of international concern (World Health Organization, 2020). Subsequently, the COVID-19 was assessed by WHO as a pandemic. The pandemic resulted in public health interventions to limit the spread and transmission of the novel coronavirus, causing significant effects on the delivery of physical healthcare procedures worldwide. For instance, to slow the spread of disease by stopping chains of transmission of COVID-19 and preventing new ones from appearing, social and physical distancing measures are strongly recommended globally, which resulted in dramatic reductions of in-person visits of patients to clinics or professionals. As this unprecedented crisis is likely to last for a long time and will possibly have multiple waves until a vaccine is available, rapidly seeking and developing a global solution to address this issue (i.e., physical distancing restrictions) will build confidence in delivering healthcare services either remotely or in-person while observing physical distancing.

Intelligent robotic systems, especially telerobotic systems, can play a positive role in this pandemic as they can effectively improve the *fully remote* or *physical distancing-aware* healthcare procedures (Tavakoli et al., 2020). Specifically, robotic and telerobotic systems can significantly reduce the risk of infectious disease transmission to frontline healthcare workers by providing a way to triage, evaluate, monitor, and treat patients from a safe distance. Moreover, medical robots have inherent advantages including steady-hand, accuracy, motion scaling, and biomotion compensation, which lead them to be able to provide general support for patients and medical professionals and further alleviate the non-COVID-19 burden placed on healthcare systems during this crisis. In fact, given the growing demands for remote-based healthcare services in the age of COVID-19, a motivation to urgently develop and apply technologies for robotics-assisted surgery has emerged. Inspired by the abovementioned points, this paper focuses on a survey addressing the subject of teleoperation on medical applications.

Teleoperation naturally indicates operating at a distance, which can perfectly meet the requirements of fully remote or in-person distancing-aware healthcare services during the COVID-19 pandemic. Meanwhile, a medical telerobotic system is capable of extending the human capabilities such as the facilitation of motion and/or force scaling, offering advantages in minimally invasive surgery including repeatability, accuracy, dexterity, fine manipulation, etc. In a general single-master/single-slave medical telerobotic system, the human applies a force on the master consoler, which results in movement commands transmitted to the slave manipulator that in turn mimics the human's operations.

Designing a teleoperation system requires addressing many issues concerning sensors and actuators, communication media, time-delay problem, stability, and transparency. Most of the relevant work and surveys focused on teleoperation, which is assumed to include a stable environment on the slave site, aiming for perfect transparency, system stability, and solving time-delay-induced problems (Hokayem and Spong, 2006; Passenberg et al., 2010). However, a survey addressing problems for teleoperation with a moving environment is seldom studied. Differing from the most researched issues such as time-delay, stability, and transparency, the most critical problem for teleoperation with a moving environment is to synchronize the slave robot's motion with the movement of the object (the environment) so that the automatic robotic motion compensations can be deployed instead of manual ones by the human operators.

A typical application of teleoperation in the medical area is manipulating with physiological organ motion caused by cardiac and respiratory activity. The cardiac motion has important local effects on the heart and areas proximal to the heart. Respiratory motion affects the movement for the majority of the body, from the thorax to the abdomen (including heart, lungs, liver, pancreas, and kidneys), and from inside to outside (such as chest and breast) of the body. It has been reported that organ displacements may range from 10 to 40 mm in anterior–posterior, left–right, and superior–inferior directions during normal breathing (Keall et al., 2006). The physiological organ motion has significant effects on medical procedures such as (i) inside or outside moving-organ surgeries (the surgeon has to manually compensate for the organ motion), (ii) moving-organ evaluation (the ability to define accurate target volumes in radiation oncology is difficult), and (iii) image-based diagnosis and monitoring (image quality and quantitative accuracy are highly effected) (Uchinami et al., 2019). When the telerobotic systems (Ballantyne, 2002) are used for the applications requiring automatic organ motion compensation, the current medical performance may need to be improved.

Among many medical telerobotic systems (Avgousti et al., 2016; Evans et al., 2018), da Vinci® robot (Intuitive Surgical Inc.) is currently the most widespread robotic surgical system, which not only can be used for teleoperation over remote distance but also can perform a variety of surgeries, evaluations, diagnosis, and monitoring. Those functionalities involve scaling the operator's actions over a small distance and with a negligible communication delay. Experimentally, the communication delay will be kept within 5–10 ms, and the effects caused by communication delay is trivial and can be negligible. In the short-distance applications, the master console and the slave manipulators are generally placed in the same operating room or different operating rooms in the same clinic; the time-delay problem, therefore, is trivial and negligible.

In this paper, we narrow down the teleoperation systems to short-distance medical telerobotics with applications accompanied by physiological organ motion, and mainly focus on the issue of motion compensation. The aim of this survey is to present the state of the art of the medical telerobotic systems with applications requiring motion compensation and the related control strategies. The rest of the paper is organized as follows. Section Teleoperation for Organ Motion Compensation deals with the control strategies of robotic-assisted systems with an emphasis on the control for automatic motion compensation. Section Applications focuses on clinical applications with telerobotic systems and solutions to effectively deliver healthcare services during the COVID-19 pandemic. Section Discussions and Future Directions discusses the perspectives of future work and concludes the paper.

## Teleoperation for Organ Motion Compensation

The mission of advancing medical telerobotic systems is to boost medicine performance by improving patient care, expanding access to high-quality therapy, and enhancing physician education, safety, and efficiency. For medical telerobotic systems with physiological organ motion such as respiratory and heartbeat motion, to minimize the risks of tool-tissue collision and tissue injury, an idea of automatic synchronization of the movement of robotic manipulator's end-tip with the moving organ is proposed. This inspires the development of telerobotic systems to provide compensation for the physiological organ motion to assist the human in performing operations accurately and safely. Indeed, if the robotic system can move a surgical instrument (e.g., catheter, ultrasound probe, forceps) in synchrony with the target tissue while the organ moves, it can provide significant benefits to the surgeon and give him/her a feeling of performing surgery on a stationary organ.

### Teleoperation Systems

In a telerobotic system, the master console controls a remote slave robotic manipulator by sending position/velocity commands and receiving potential haptic/visual feedback signals, as well as the information of slave robot status. Teleoperation systems can be divided into three categories with their features: unilateral teleoperation systems, bilateral teleoperation systems, and multilateral teleoperation systems.

In a typical single-master/single-slave teleoperation system, if the slave does not possess a force sensor, which causes the human operator losing the sense of touch, then this system is called a unilateral teleoperation system. In contrast, if the slave possesses force sensors and is able to transmit the force feedback to the master, then this system is called a bilateral teleoperation system. In other words, the human can feel the interaction force between the slave robot and what it is touching, enabling the human to efficiently manipulate the master robot to provide appropriate commands. When a teleoperation system consists of more than one master console and/or slave manipulator and involves more than one sensed and command signal flow between the human operator and the environment, the system is called a multilateral teleoperation system. A multilateral framework not only allows for a one-to-one correspondence between the operator–master and the slave–environment sets but also realizes collaborative scenarios between multiple operator–master sets and/or multiple slave robots.

### Physiological Organ Motion

The motion of a moving organ is primarily induced by respiratory and/or heartbeat motions with different frequency ranges. In order to mimic the physiological organ motion in experiments, the studies can be classified into two categories: organ simulators and living organ. By designing mechanical devices, the organ simulators can be controlled to mimic the moving organ's motion based on pre-acquired organ motion data (Yang et al., 2016; Cheng et al., 2019) or biological signals (Cheng and Tavakoli, 2018b). *In vivo* experiments use living porcine organ (Kesner and Howe, 2014) or dog organ (Mansouri et al., 2018) to demonstrate the control techniques. Specifically, in Yang et al. (2016), a stereo video of *in vivo* porcine heart, which recorded image sequence of a totally endoscopic coronary artery bypass graft from a da Vinci (Intuitive Surgical, CA) surgical platform, was used to measure the 3D heart positions offline by vision tracking. The quasi-periodic 3D heart motion signals were transmitted to a Motoman SIA-5F (Yaskawa America, Inc., Miamisburg, OH, USA) 7-DOF serial manipulator (Cheng et al., 2019) to control the manipulator to work as a real heart organ.

### Motion Compensation Control Techniques

To compensate for the physiological organ motion and synchronize a robot's motion with the organ's motion, various control methods have been proposed for both handheld robotic systems (Yuen et al., 2009; Poulsen et al., 2012; Winter et al., 2015; Kolbitsch et al., 2018; Salehi et al., 2018; Ting et al., 2018) and telerobotic systems (Ginhoux et al., 2005; Gangloff et al., 2006; Cheng et al., 2018). In the paper, we mainly focus on motion compensation control methods for telerobotic systems, which generally falls into four categories: position control, force control, impedance control, and hybrid control.

#### Position Control

The position-based controllers need the real-time organ position and use that to synchronize the slave's movement with the organ's motion. For a teleoperation system, to further control the slave robot to mimic the human's operation, the summed positions of the master and the moving organ are used as a reference position for the slave robot to follow. A pure position-based telerobotic control system belongs to a unilateral teleoperation system as it provides the human without haptic feedback.

Before discussing robot controllers, a vital issue is to measure the moving organ position in real time. To this end, many image-based sensors have been widely used in research such as high-speed camera/laparoscopy (Nakajima et al., 2014), X-ray fluoroscopy (Ma et al., 2020), computed tomography (CT) (Su et al., 2013), magnetic resonance imaging (MRI) (Yang et al., 2014), positron emission tomography (PET) (Bettinardi et al., 2013), and ultrasound imaging (US) (Bowthorpe and Tavakoli, 2016a,b; Diodato et al., 2018). To get performance, hybrid imaging systems are also developed to measure precise organ motion including MRI/US imaging (Celicanin et al., 2018), MRI/CT imaging (Neumann et al., 2017), PET/CT imaging (Bettinardi et al., 2013; Pepin et al., 2014), and PET/MRI (Kolbitsch et al., 2018). The above-listed measurements have their advantages and limitations, which are elaborated in Table 1.

**Table 1:** Advantages and disadvantages of medical imaging measurements.

**Measurements**	**Advantages**	**Disadvantages**
High-speed camera	Accurately measure real-time organ position by tracking points on the tissue	It only visualizes the outer surface of the moving organ and is not appropriate for surgeries performed inside of the organ.
X-ray fluoroscopy and CT	Cancerous organ scanning inside the body, such as of the thoracic and abdominal viscera. Precise.	It exposes patients to a dose of radiation that is capable of damaging cells and initiating changes leading to cancer.
MRI	Avoid radiation issue and can provide high-quality imaging, especially to discover tumors.	Expensive. Patients with iron-containing metallic implants cannot undergo MRI scanning because MRI machine can dislodge those implants.
US	Study heart function, blood flow in the neck or extremities, gallbladder disease, and fetal growth and development.	Image quality is heavily operator-dependent, and its sampling frequency is low.

Non-image-based sensors have also been used to collect moving organ motion data such as sonomicrometric sensors (Bebek and Cavusoglu, 2007), optical measurement (Ruszkowski et al., 2016), and electromagnetic tracking system (Loschak et al., 2020).

Once the position of the moving organ is measured, robot controllers aimed for motion compensation can be deployed. Controllers for physiological organ motion compensation can be classified into error feedback controllers, predictive feedforward (prediction-based) controllers, and predictive feedback controllers, as elaborated in the following:

**Error feedback controllers** directly use the measured position as a reference signal for the medical robot. A proportional–integral–derivative (PID) controller is widely used to continuously calculate an error value (Murphy, 2004). However, the error feedback controller is found to be unable to reduce tracking error sufficiently if used solely.

**Prediction-based controllers** use the estimated current organ position as the setpoint to move the medical tools. It aims to develop accurate mathematical models of the organ's motion by using one or more previous measured motion dataset. The primary goal is to improve motion tracking performance by developing estimation methods. To this end, Taken's theorem (Ortmaier et al., 2005), artificial neural network (Cheng and Tavakoli, 2019b; Hirai et al., 2019), extended Kalman filter (EKF) (Liang et al., 2014), receding horizon model predictive controller (Bebek and Cavusoglu, 2007), and recursive least squares-based adaptive filter (Tuna et al., 2014) have been investigated in the developments of prediction-based controllers.

**Predictive feedback controllers** not only need the organ's current position but also take the tracking error into account. By considering the physiological organ motion as periodic disturbances, controllers such as model predictive controllers (MPCs) (Gangloff et al., 2006; Vrooijink et al., 2017), Smith predictor-based controllers (Bowthorpe et al., 2013; Bowthorpe and Tavakoli, 2016a), generalized predictive controllers (GPCs) (Bowthorpe and Tavakoli, 2016b), and repetitive-GPC (R-GPC) (Ginhoux et al., 2005) were used. As these methods rely on the known organ motion model, the robustness of the system to irregular organ motion is challenging.

#### Force Control

For applications that require tool-tissue contact such as ablation and biopsy with controlled depth, a significant breakthrough in medical telerobotics is facilitated by force-reflecting haptic feedback, which allows the human to perceive the forces applied by the slave robot on the environment (a moving target). Force feedback (haptic feedback) increases the transparency of the teleoperation, which enhances human operator's immersive maneuver on the master consoler. Such function, as mentioned earlier in the paper, requires sensory feedback information. In other words, a force/torque sensor should be mounted on the slave side to measure and transmit slave–environment interaction forces. To simultaneously compensate for the physiological organ motion, various force-based control methods are proposed. The control goal is to keep the slave–environment interaction forces at a constant value so that the human can have a haptic feeling that the environment is motionless through force feedback.

Moreira et al. (2014) proposed a force control scheme using an active observer (AOB) based on a viscoelastic interaction model (soft tissue model) to compensate for the physiological motion. The model-based force control used the AOB to estimate the system states and an extra state, which is employed to compensate for system disturbances and modeling errors. Dominici and Cortesão achieved motion compensation by designing a cascade MPC architecture with a Kalman AOB (Dominici and Cortesão, 2014). The AOB inner loop provides stable closed-loop dynamics, and the MPC outer loop generates reference forces for AOB control for autonomous motion compensation. The authors further proposed another force control scheme by using a double AOB architecture (Cortesão and Dominici, 2017). In the work of Mohareri et al. (2014), the authors developed an asymmetric force feedback control system for bimanual telerobotic surgery using the da Vinci surgical system. To avoid instability issues caused by the closed-loop system, the authors proposed to use one hand to exert force through the master robot and use the other hand to perceive force feedback from the slave robot. He et al. (2019) proposed a neural network-based force control scheme to compensate for the eyeball motion in retinal surgery, in which the tool–eyeball interactive force is fed into the neural network and the latter is trained to command the robot manipulator to move according to the eyeball movements.

#### Impedance Control

Different from the position control and the force control, which are utilized to control position or force variables separately, impedance control is a compliant control, which is employed to achieve desirable dynamic interaction between a robot manipulator and its environment. In other words, impedance control can control the dynamic relationship between robot motion and robot–environment interaction force as desired. For a robotic manipulator aiming to compensate for the organ's motion, the robot and the moving organ can be expressed as impedance and admittance, respectively (Hogan, 1984, 1985). The goal of impedance control is to regulate the dynamic relationship to achieve the requirements of automatically compensating for the organ motion while keeping the interaction force in a safe range.

Florez et al. (2012) proposed a method that uses an impedance control on a handheld robotic instrument to compensate for physiological motion. The handheld system allows the human to perform low-frequency motions that correspond to the task. At the same time, the part of the instrument contacting the moving organ actively moves in synchronism with the organ's motion to keep a constant contact. Zarrouk et al. (2010) proposed an adaptive control architecture based on model reference adaptive control to solve the 3D physiological motion compensation in beating-heart surgery. A reference impedance model and an adaptive controller were designed for the surgical robot. The aforementioned impedance-controlled systems are developed for handheld medical robotics instead of teleoperated systems. In the work of Cheng (Cheng and Tavakoli, 2018a; Cheng et al., 2018, 2019) and Sharifi et al. (2018), the model reference adaptive control was applied to the bilateral teleoperation systems separately. The authors designed two reference impedance models for the master and slave robots, respectively. The slave reference impedance model was used to make the slave robot compensate for the living organ's motion, while the master reference impedance model has the ability to ensure the human to perceive non-oscillatory robot–organ interaction force. The oscillatory haptic feedback caused by oscillatory motion and force sensor inertia is filtered out by the master reference impedance model.

#### Hybrid Control

Hybrid control combines two or more control schemes together, including hybrid position/force control, hybrid position/impedance control, etc. The goal of hybrid control is mainly to develop a compliant control scheme to achieve specific task requirements. In Yuen et al. (2010) and Kesner and Howe (2014), the authors separately incorporated position control and force control to achieve beating-heart motion compensation. These methods combined the US guidance with a force controller and are aimed to incorporate a feedforward term that contains the estimated motion of a beating heart. The US was used to measure the position of the moving organ, while the force controller was utilized to extend the device application from free motion to constrained contact motion. Nakajima et al. (2014) used visual servoing to compensate for the organ motion and performed haptic feedback using an acceleration-based bilateral control method. System stability was evaluated through frequency characteristics and root locus. In Cheng and Tavakoli (2018b), an impedance control combined with an US image-guided position control was developed in a teleoperation system. The US scanner estimated the moving organ position and transmitted it along with the master robot position to the slave robot as a position reference. For non-oscillatory haptic feedback, a reference impedance model was designed for the master robot to provide the human with a steady slave–organ interaction force.

## Applications

**Figure 1:**
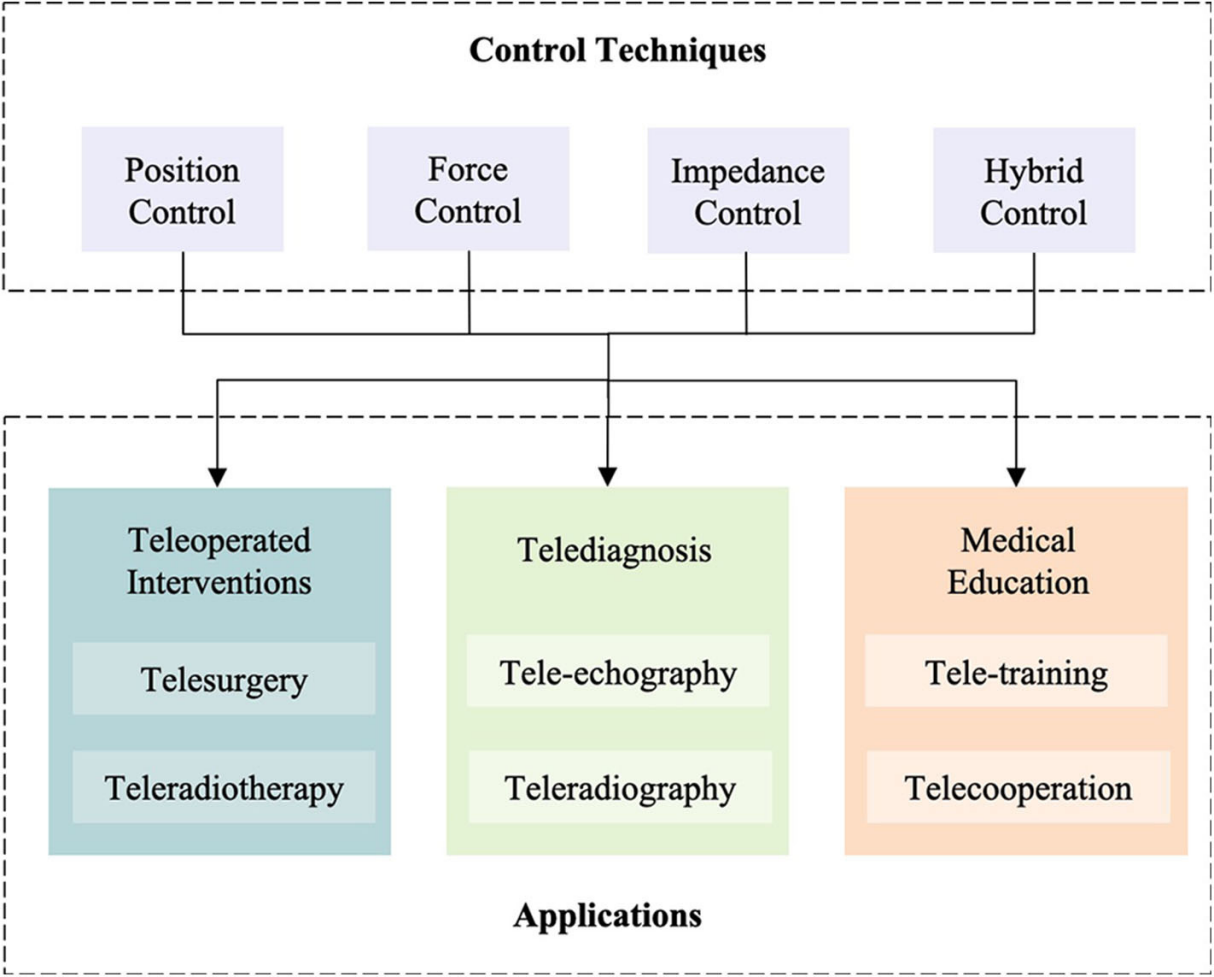
Scheme of the control methods for physiological motion compensation and the potential applications.

Significant interest has been documented for both interventional (e.g., therapeutic treatments such as surgery and protontherapy) and diagnostic (e.g., US scan, X-ray scan, and biopsy) applications (Figure 1).

The corresponding telerobotic systems can significantly reduce the risk of infectious disease transmission to frontline healthcare workers by making it possible to evaluate, monitor, and treat patients from a safe distance. Moreover, the teleoperation techniques are able to provide general support for patients and medical professionals, alleviating the non-COVID-19 burden placed on healthcare systems during this crisis. The latter, i.e., secondary prevention and disease management of non-COVID-19 individuals who need therapeutic treatments or diagnosis during this difficult time, is of equal importance. Telerobotic and autonomous systems can support healthcare staff such as physiotherapists and surgeons during the COVID-19 pandemic through facilitating fully remote or in-person distancing-aware physical treatments and diagnosis services.

### Teleoperated Intervention

#### Telesurgery

Telerobotics applications mostly involve articulated robot configurations with an interchangeable surgical tool that is mounted on the end effector of the slave robot (surgical robot). Robot systems have been developed from the first functional telesurgery system—ZEUS—to the da Vinci surgical system; the latter is currently the only commercially available surgical robotic system. However, telerobotics for applications with physiological organ motion are mostly in the domain of research yet.

Most of the proposed systems are application-specific medical telerobots, such as the telerobots used for beating-heart surgery (mitral valve prolapses and repair, atrial septal defect, atrial fibrillation) and percutaneous nephrolithotomy surgery (kidney stones, kidney cysts, kidney blockage). Compared to conventional surgery operations, the surgeries assisted by telerobotic systems requiring organ motion have significant advantages. First, the master–slave system enables remote or physical distancing-aware surgical procedures during the COVID-19 pandemic. Second, automatic compensation for complex physiological organ motion greatly reduces the difficulty of operation for surgeons and increases surgical accuracy, which turns to improve patient safety. Third, advanced technique introduces minimally invasive robotic surgery, which can be used for the surgeries mentioned above with benefits including small incisions, little pain, low risk of infection, short recovery time, and reduced blood loss. Last but not least, specifically for beating-heart surgery, robotic surgery has been found to have additional advantages over the conventional arrested-heart surgery; the latter has to employ a heart–lung bypass machine (Angelini et al., 2002).

#### Teleradiotherapy

When a tumor locates close to the vital organs (heart, lung, etc.), radiation therapy is generally recommended as a useful treatment to destroy cancer cells and slow tumor growth without harming nearby healthy tissue. The goal of radiation therapy is to assess the true volume of the tumor and its real motion and to obtain an accurate target delineation and an accurate and personalized definition of the treatment plan (Khan and Gibbons, 2014). As the breathing-induced motion has significant effects on organs (e.g., liver, lung, breast, kidney, prostate, and pancreas), radiation therapy, accurately and automatically compensating for continuous physiological respiratory motion of organs, is necessary. Indeed, if not correctly compensated, organ motion can lead to a spreading of the thermal dose, which is the cause of two severe issues: (i) loss of treatment efficiency, and (ii) generation of unplanned lesions in adjacent healthy tissues. Moreover, teleradiotherapy will be useful to get rid of the side effects of radiation therapy to the physicians.

### Telediagnosis

#### Tele-Echography

Ultrasound is an imaging modality that plays a significant role in medical emergency and surgical decision diagnosis. To compensate for the limited availability of ultrasound experts in isolated areas (such as physical distancing-aware caused by COVID-19 pandemic), the use of robotic telemedicine systems is gaining attention. A commercial MELODY tele-ultrasound robotized system was developed by AdEchotech SME (France) (Vieyres et al., 2013) for long-distance US diagnosis. The slave robot is attached to an US probe through a probe holder. The human at the master site moves a fictive US probe as required for an echographic diagnosis. The MELODY system was designed to fulfill remote static organ diagnosis without considering issues such as moving organ motion compensation. Sharifi et al. (2017) developed a bilateral telerobotic system for echography in beating-heart surgery. Although it is just a proof of concept, the idea of the control scheme is worth to be considered for future commercial popularizing.

#### Teleradiography

Teleradiography allows radiologists or physicians to provide services without physically being at the location of the patient. Similar to tele-echography, by mounting the CT scanner or X-ray holder on the slave robot, the radiologists or physicians can remotely diagnose the patient's body without being exposed to radiation. Most importantly, robot-assisted organ motion compensation will be a benefit for accurate imaging and preventing the over-radiation of the patients.

### Training and Education

The wide applicability of teleoperated interventions and telediagnosis will depend on not only the maturity of the technology but also the skill level of trained physicians. These applications require specialized skills compared to traditional methods. Moreover, it is essential that medical schools are equipped with such technologies to appropriately train physicians. Existing possibilities include the use of multilateral teleoperation systems with a multiple control console configuration to enable training or collaborative medical applications (Shahbazi et al., 2018; Cheng and Tavakoli, 2019a).

## Discussions and Future Directions

Influenced by the COVID-19 pandemic, the presented review focuses on a potential solution for remote and physical distancing-aware healthcare delivery—medical telerobotics. The review studies the medical telerobotics for applications with physiological organ motion, and discusses control schemes for motion compensation, potential applications, and associated benefits. The medical telerobotics have been already employed in a wide range of diagnostic and interventional applications in different medical disciplines. To successfully apply medical telerobotic technologies to clinical practice, a significant issue is to develop appropriate control schemes for the specific application.

Solutions only involving visual servoing (image-based position control) are found to have several limitations: (i) artificial and natural landmarks occlusion will affect the measurements of the landmark-based sensors, (ii) tissue deformation during contact tasks will affect organ position measurement, and (iii) physiological motion induces oscillatory force feedback and will affect human's performance. To deal with those issues, a latest research proposed a novel printing procedure to fabricate an electrical-impedance-tomography strain sensor on an ex-vivo breathing lung. The authors integrate a visual sensing system with a 3D printer to track the time-varying 3D geometry of the lung (Zhu et al., 2020). The method presented in the abovementioned research could aid modern medical treatments in myriad ways, such as printing electrode arrays for neural interfaces and printing bioscaffolds with engineered cells for tissue regeneration.

Another issue of position control is that when a position controller is used for contact tasks, the contact constraints will be treated as a disturbance resulting in increasing position tracking error, which probably leads to excessive interaction force. Therefore, a position control scheme in position tracking task works better in free motion than constrained motion. Applications such as biopsy and percutaneous puncture are more suitable to be performed by a position-based telerobotic control scheme.

In clinical practice, most medical interventions require direct interaction between one or more tools with the patient's organs. To assure the patient's safety and provide the surgeon with a comprehensive perception, force feedback is necessary for medical telerobotics during robotized interventions. In addition to the limitations of sensors and systems available for force feedback teleoperations, the force control scheme has its drawbacks as well. As the goal of pure force control is to keep the contact force as the reference without position limitations, it leads to force control working in constrained motion.

In fact, both position control and force control can be treated as extreme situations of impedance control. Specifically, the position controller has infinite impedance, while the force controller has zero impedance. Those controllers may be appropriate for applications in which the work exchanged between the robot and its environment is negligible. For applications where power exchange cannot be ignored, hybrid position/force interaction control or impedance control can be used to provide techniques to accommodate the side effects.

Medical telerobotics for applications requiring physiological organ motion have been developed considerably for the last 20 years, and they will be necessarily developed much further in the coming years, especially in the field of surgery and diagnosis. However, further efforts are required to address both clinical and technological challenges.

An existing difficulty for the adoption of medical telerobotic technologies is to deliver accuracy and precision medical procedures, which require particular effort to overcome. For instance, precise interaction and force applied on the tissue by the robotic instrument, accurate dose delivery to the patients, and limited radiation exposure of the patients should be strictly controlled according to specific medical practice. Considering and regulating applicable requirements and specifications for medical procedures and devices will be a benefit for bridging the gap between engineering and medicine. Both patients' and physicians' safety are always the priority in clinical practice. Therefore, when adopting a medical device in clinical practice, the stability of the system and the robustness and reliability to an unforeseeable emergency such as irregular organ motion should be of great concern.

Another significant issue that limits the spread of telerobotic system in clinical practice is the high cost of the medical devices. As the system development requires interdisciplinary knowledge including medicine, engineering, computer science, and mathematics, the challenges and costs are doubtless high. As a result, mature technology and standard requirements of specifications would be beneficial.

Ultimately, medical telerobotics is a promising technology, which has significant advantages for healthcare delivery and can play a positive role in the COVID-19 pandemic as it can effectively improve the remote or physical distancing-aware healthcare procedures. The present review study of teleoperation for medical applications requiring physiological motion shows that various control methods have been proposed for specific applications. As a result, preliminary research toward this direction has already been achieved, but the deep potential of medical telerobotics for applications requiring organ motion remains largely unexploited.

## Author Contributions

LC completed the work including literature review and preparation for the initial draft of the manuscript. MT provided guidance and valuable suggestions/discussions and was involved in the editing of the manuscript. All authors contributed to the article and approved the submitted version.

## Conflict of Interest

The authors declare that the research was conducted in the absence of any commercial or financial relationships that could be construed as a potential conflict of interest.
